# Differential Virulence and Host-Specific Fitness of Regionally Distinct Human-Derived Powassan Virus Lineage 2 Strains

**DOI:** 10.4269/ajtmh.24-0776

**Published:** 2025-05-13

**Authors:** Rachel E. Lange, Julia C. Pritchard, Anna S. Jaeger, Tyler D. Bold, Jennifer L. White, Amy B. Dean, Rene C. Hull, Kirsten St. George, Alan P. Dupuis, Matthew T. Aliota, Alexander T. Ciota

**Affiliations:** ^1^ Arbovirus Laboratory, Wadsworth Center, New York State Department of Health, Slingerlands, New York;; ^2^ Department of Biomedical Sciences, State University of New York University at Albany School of Public Health, Albany, New York;; ^3^ Department of Veterinary and Biomedical Sciences, University of Minnesota Twin Cities, St. Paul, Minnesota;; ^4^ Division of Infectious Diseases and International Medicine, Department of Medicine, Center for Immunology, University of Minnesota Medical School, Minneapolis, Minnesota;; ^5^ Bureau of Communicable Disease Control, New York State Department of Health, Albany, New York;; ^6^ Laboratory of Viral Diseases, New York State Department of Health, Albany, New York

## Abstract

Powassan virus (POWV; family Flaviviridae) is a tick-borne encephalitic virus endemic to Canada, the United States, and Russia. In the United States, POWV is transmitted by ixodid ticks, and transmission foci reflect the geographic range of these vectors, primarily *Ixodes scapularis*. Thus, northeastern and midwestern regions of the United States contain the highest human case burdens and prevalence of infected ticks. Notably, New York (NY) and Minnesota (MN) have a long history of POWV transmission to humans. Over time, genetic divergence has occurred in these regions, giving rise to distinct midwestern and northeastern clades. Despite the established circulation of POWV, increases in reported human cases, and documented genetic distinction, an understanding of strain-specific POWV virulence is limited because of the lack of human isolates. In 2020 and 2021, two POWV strains were isolated from fatal human cases from MN (deer tick virus [DTV] MN-PV320) and NY (DTV NY21-027). Here, we provide the first characterization of geographically distinct, contemporary, human POWV isolates. Comprehensive genetic characterization was completed and phenotypic variability was determined in vitro and in vivo . Although strain fitness was similar in *I. scapularis*, higher mortality rates were measured in a susceptible POWV mouse model after infection with DTV NY21-027 compared with DTV MN-PV320. Genetic analysis revealed several variable amino acid substitutions, including I2173L in DTV NY21-027, which was selected for in all strains after neurological infection. These data suggest that genetic divergence of POWV strains from regionally distinct transmission foci could contribute to strain-dependent pathogenic potential in humans.

## INTRODUCTION

Powassan virus (POWV; family Flaviviridae) is the sole member of the tick-borne encephalitis serogroup in North America.^1^^,^^2^ In the United States and Canada, POWV exists in two genetically distinct lineages that are maintained in discrete ecological cycles with some overlap.^3^ Prototype POWV or Powassan virus lineage 1 (POWV-1) is maintained between *Ixodes cookei* and *Ixodes marxi*, with groundhogs and squirrels as putative reservoir hosts.^3^ POWV lineage 2 or deer tick virus (DTV) is maintained in a cycle between *Ixodes scapularis *and small mammalian hosts, with shrews currently recognized as the most likely reservoirs.^3^^,^^4^ Geographic range of these Ixodid ticks reflects endemic regions for POWV (*I. cookei* and *I. marxi* for POWV-1 and *I. scapularis* for DTV), primarily concentrated in the northeastern and midwestern United States and southeastern Canada. Most human cases are assumed to be a result of DTV infection because of the aggressive generalist-biting behavior of *I. scapularis* compared with the burrow-dwelling, host-specific *I. cookei* and *I. marxi*.

Human infection follows a biphasic course of disease characterized by nonspecific febrile illness during acute infection and progression to neurological involvement after breaching the blood–brain barrier and infecting the central nervous system.^1^^,^^2^ Common symptoms during acute infection include fever, fatigue, and general weakness, whereas neuroinvasive disease is often associated with confusion, focal neurological deficits, meningoencephalitis, headache, seizures, and hemiplegia.^5^ Although there is a reported 88% survival rate from acute infection, about a third of survivors display long-term neurological sequelae.^5^ Clinical diagnosis is largely reliant on serological assays in which POWV-1 and DTV are indistinguishable; therefore, determining the causative lineage requires isolation and sequencing of virus. Isolation of virus from human infection is rare because of low transient viremia and lack of cerebrospinal fluid (CSF) or brain tissue postmortem.^5^ To date, the understanding of POWV pathogenesis is limited to reported human cases, fewer than 10 sequenced human-derived strains, and studies in animal models using historic high-passage isolates that may not be generalizable.

The first human case of POWV was identified from a pediatric encephalitis case in Ontario, Canada in 1958.^6^ Subsequently, cases were reported in New Jersey, New York State (NYS), and Canada.^7^^–^^9^ In 1994, 1999, and 2000, cases were reported in Massachusetts, Vermont, and Maine, respectively.^10^^–^^12^ Because DTV was not identified as a genetically distinct POWV lineage until 1997, all cases were presumed to be because of POWV-1 until the first confirmatory case of DTV in 2001.^13^^,^^14^ After 2001, substantial increases in POWV were reported in the northeastern United States, and transmission foci in the midwestern United States emerged with confirmed cases in Michigan, Wisconsin, and Minnesota (MN) from 2002 to 2008.^11^^,^^15^^,^^16^ Overall, yearly POWV cases have increased from 0.7 cases/year from 1958 to 1998 to 12 cases/year from 2004 to 2012 and 33 cases/year from 2013 to 2023.^17^ NYS, MN, Wisconsin, and Massachusetts now account for the highest case burdens.^17^ Currently, NYS and MN remain two highly endemic regions with increasing numbers of human POWV cases, prevalence of infected *I. scapularis* (1–6% positivity in questing field-collected ticks), and human seroprevalence (1–10%).^11^^,^^18^^–^^20^

Geographic spread and establishment of POWV throughout the United States mirrors the spread of tick-borne encephalitis virus (TBEV) across Eurasia. Although tick-borne bacterial pathogens like *Borrelia burgdorferi*, the causative agent of Lyme disease, spread diffusely across geographic ranges with contributions from vertebrate reservoirs, POWV and TBEV are maintained in highly focal transmission hot spots that are spatially and temporally stable.^21^^–^^23^ Vertical transmission and cofeeding may play prominent roles in POWV and TBEV ecological maintenance.^24^^–^^26^ In Eurasia, virus establishment in regionally distinct foci and geographic separation resulted in the genetic divergence of TBEV into three main subtypes (seven total).^27^ These subtypes, which are 10–20% divergent at the nucleotide level, have different virulence phenotypes that manifest as different pathologic outcomes in human cases.^27^^,^^28^ For example, the Far East TBEV subtype (i.e., Russian spring summer encephalitis virus) causes severe meningoencephalitis in 20–40% of patients, whereas the Central European TBEV subtype is characterized as a mild febrile illness, with less than 2% of cases progressing to neuropathy.^29^ Nucleotide divergence between the northeastern and midwestern clades of DTV is 5–10%, yet lack of characterization of contemporary strains, underreporting of human cases, and limited access to human-derived isolates have contributed to a gap in knowledge regarding the relationship between genetic variability, viral fitness, and clinical presentations.^14^^,^^23^^,^^30^

This study aimed to determine if two DTV strains associated with fatal human infections from two spatially distinct endemic regions, NYS and MN, display phenotypic variability in virulence and viral fitness in a susceptible mouse model, cell culture, and *I. scapularis*. Significant phenotypic differences were identified that are important for understanding the current landscape of POWV diversity and virulence across established transmission hot spots in North America.

## MATERIALS AND METHODS

### Cells and viruses.

Baby hamster kidney cells (BHK-21, ATCC, and CCL-10) were grown in minimal essential media (MEM; Gibco, Invitrogen, Carlsbad, CA) supplemented with 10% heat-inactivated fetal bovine serum (FBS; Hyclone, Logan, UT) and maintained at 37°C and 5% CO_2_. Human neuroblastoma cells (SH-SY5Y, ATCC, and CRL-2266) were grown in a 1:1 mixture of Eagles minimum essential media (Gibco, Invitrogen) and Ham’s F12 media (Gibco, Invitrogen) and maintained at 37°C and 5% CO_2_. FBS (10%) was added to SH-SY5Y media to encourage differentiation as previously described.^31^ Confluent monolayers were generated by seeding six-well tissue culture plates (STEMCELL Technologies, Seattle, WA) with 1.0 × 10^6^ cells per well and maintaining them for 3 and 5 days before experimentation.

Viral isolates POWV-1 LB and DTV R59266 were provided by the CDC. POWV-1 75-1702, POWV-1 63-002, and DTV 18071-054 were identified and collected through New York State Department of Health (NYSDOH) tick surveillance and testing.^32^^,^^33^ DTV NY21-027 and DTV MN-PV320 were detected by reverse-transcriptase polymerarse chain reaction (RT-PCR) through NYS and MN department of health diagnostic laboratories and isolates generated from human patient cerebrospinal fluid (CSF).^18^ Briefly, RT-PCR was run as previously described under the following thermocycler conditions: 15 minutes at 48°C and 2 minutes at 95°C followed by 45 cycles of 3 seconds at 95°C and 30 seconds at 60°C.^34^ Tick- and human-derived strains generated at the NYSDOH were identified by a multiplex quantitative (q)RT-PCR as previously described.^18^ After confirmation by qRT-PCR for POWV RNA, tick homogenates or human CSF samples were amplified on BHK-21 cultures twice for a passage history of “B2.” After observation of cytopathic effects between 5 and 7 days postinfection (DPI), supernatant was collected, clarified, and stored at −80°C in BA-1 media (M199 medium with Hank’s salts, 1% bovine albumin, tris(hydroxymethyl)aminomethane, sodium bicarbonate, and antibiotics) with 20% heat-inactivated FBS.

### Mice and ticks.

C57BL/6 mice were purchased from Jackson Laboratories (Bar Harbor, ME). Six-week-old mixed-sex mice were used for all experiments. This study was approved by the University of Minnesota Institutional Animal Care and Use Committees (Protocol no. 2102-38855A). All animal procedures with POWV were conducted in animal biosafety level 3 facilities at the University of Minnesota, Twin Cities.

The following reagent was provided by the CDC for distribution by BEI Resources, National Institute of Allergy and Infectious Diseases, NIH: *I. scapularis* nymph (live), NR-44116. All ticks were maintained at 95% relative humidity (RH) and 20°C on a 16:8 light:dark cycle. Ticks were stored in 2-ounce glass specimen jars with a bottom layer of Plaster of Paris or in 7-mL ventilated cell culture tubes (ThermoFisher Scientific, Waltham, MA). All tick procedures with POWV were conducted in arthropod biosafety level 3 facilities at the Wadsworth Center Arbovirus Laboratory.

### Subcutaneous inoculation.

Mice were inoculated under isoflurane anesthesia in the left hind footpad with 3 log_10_ plaque-forming units (PFU)/mL of human-derived strains DTV NY21-027 and DTV MN-PV320, tick-derived strain DTV 18071-054, or sterile phosphate buffered saline (PBS) to serve as experimental control. A total of 39 mixed-sex mice were randomized into four treatment cohorts and a mock cohort. Submandibular blood draws were performed on one half of each group 1, 3, and 5 DPI, and they were performed at 2, 4, and 6 DPI for the other half of each group; additionally, they were performed immediately before euthanasia for each mouse. Serum was collected to verify viremia and compare replication kinetics. Mice were weighed daily and monitored for clinical signs of morbidity. Morbidity was rated on a scale of zero to six: zero: normal; one: ruffled fur; two: hunching, mild to moderate weakness, ataxia, distended abdomen, and diarrhea; three: severe weakness, tremors, circling, head tilt, and seizures; four: paralyzed limb; five: morbid and nonresponsive; and six: dead. Mice were euthanized at the experimental end point of 21 DPI or if they met early end-point euthanasia criteria as defined by a loss of 20% of original body weight or a clinical score of greater than or equal to three.

### Mouse necropsy.

After inoculation with POWV or PBS, mice were killed at 21 DPI or if they met early end-point criteria. Immediately after euthanasia, brain tissue was harvested, rinsed with PBS, and stored in PBS supplemented with 20% FBS and 1% antibiotic–antimycotic solution. These tissues were then weighed and homogenized by 5-mm stainless steel beads with a TissueLyser (Qiagen, Germantown, MD) and used for onward analysis of infectious virus and viral sequencing. Briefly, homogenized samples were clarified by centrifugation at 10,000 × *g *for 2 minutes before any further analysis.

### Sequencing.

Whole-genome sequencing was conducted as previously described.^33^ Briefly, RNA was extracted from respective samples (cell culture supernatant, tick homogenates, brain homogenates, and serum) using an automated nucleic acid extraction kit and associated instrument (ThermoFisher Scientific) or manually using the Directzol RNA Extraction Kit according to the manufacturer’s instructions (Zymo Research Corporation, Tustin, CA). Extracted RNA was used to generate six overlapping fragments (∼2 kb) (Supplemental Table 1) that span the full genome using a Superscript III One-Step RT-PCR kit (ThermoFisher Scientific). Products were visualized on a 1% agarose gel and purified using a Zymo DNA Clean and Concentrator 5 ng Kit (Zymo Research Corporation), and fragments were pooled at equal molar concentrations.^33^ Next-generation sequencing was carried out at the Wadsworth Center Advanced Genomics Technologies Core using an Illumina MiSeq platform (2 × 500-bp paired-end reads; Illumina, San Diego, CA).

Sequence analysis was carried out in Geneious Prime 2021.2. Reads were paired and merged, primers were trimmed, and they were mapped to a lineage-specific reference (POWV-1: POWV-1 LB, NC003687; DTV: DTVWiA08, HM440560.1). Consensus sequences were generated based on a 65% minimum frequency threshold for nucleotide calling. Reads were then mapped to their respective consensus sequence, in which intrahost small nucleotide variants (iSNVs) were identified based on a 2% minimum frequency threshold. Only the coding regions of each strain were used for further analysis. Phylogenetic trees, nucleotide mutations, and amino acid substitutions were identified based on multiple alignment using fast Fourier transform alignments and crossreferenced to all publicly available strains (*n* = 441) on the “Powassan-genomics” NextStrain page.^35^^,^^36^ Strains used in these analyses include mammal- and tick-derived strains, of which less than 10 strains with full-genome sequences available were human derived. Phylogenetic trees were generated by using the Geneious Tree Builder based on neighbor joining trees, Tamura–Nei distances, and 1,000 bootstraps and visualized in iTOL: Interactive Tree of Life v. 6. Viral diversity was determined by the proportion of iSNVs to the number of nucleotides in the coding region of the POWV genome. To calculate an accurate estimation of genome-wide diversity, analysis was limited to regions with sufficient sequencing depth (minimum of 30 reads per base), including the E, NS2a, NS2b, NS3, NS4a, and NS5 genes. Challenge stocks are defined by the B2 cultures used as inputs for all experiments (sequenced after passage and before experimentation), whereas brain and serum outputs are defined as the live virus harvested from the respective organ postmortem.

### Plaque assay.

Plaque assays were carried out as previously described.^33^ Briefly, confluent monolayers of BHK-21 in six-well tissue culture plates were inoculated with 0.1 mL of sample in duplicate. After a 1-hour adsorption period at 37°C and 5% CO_2_, a 3-mL overlay of MEM, 10% heat-inactivated FBS, and 0.6% oxoid agar was added to each well. Plates were incubated at 37°C and 5% CO_2_ for 3 days followed by the addition of a second overlay made up of MEM, 2% FBS, 0.6% oxoid acid, and 2% neutral red (Sigma-Aldrich, St. Louis, MO). Plates were incubated overnight with the second overlay, and plaques were counted the next day. Viral titers were expressed as PFU per milliliter.

### In vitro growth kinetics.

Viral growth kinetics were carried out in confluent monolayers of BHK-21 and SH-SY5Y cultures. Cells were seeded at 1 × 10^6^ cells per well and allowed to grow to confluency for 3 and 5 days, respectively, in six-well tissue culture plates. Confluent monolayers were inoculated in triplicate with 0.1 mL of sample at a multiplicity of infection of 0.01 PFU/mL. After adsorption for 1 hour at 37°C and 5% CO_2_, the inoculum was removed, wells were washed three times with 2.0 mL of PBS, and 3.0 mL of the appropriate maintenance media were added. Supernatant was collected at 24-hour intervals up to 120 hours postinfection (HPI). Plaque titration on BHK-21 cells was used to determine viral load.

### Synchronous infection of ticks and viral detection.

Nymphal ticks were experimentally infected by immersion as previously described.^33^^,^^37^ Briefly, nymphs were held at reduced RH (65%) for 48 hours before immersion in 1 × 10^6^ PFU/mL of virus. After incubation at 34°C for 1 hour, nymphs were chilled, washed twice with PBS, and dried. Nymphs were held at 95% RH and 20°C on a 16:8 light:dark cycle until processing at 7, 14, 21, and 28 DPI, at which time they were individually sorted and frozen at −80°C.

To test individual ticks for viral detection and estimated viral load, 5-mm stainless steel BBs (Daisy Outdoor Products, Roger, AR) and 0.6 mL of diluent (20% heat-inactivated FBS in Dulbecco PBS, 50 µg/mL penicillin/streptomycin, 50 µg/mL gentamicin, and 2 µg/mL fungizone; Sigma-Aldrich) were added to each tube. Nymphs were homogenized using a Retsch Mixer Mill, MM 301 (Retsch, Newtown, PA) at 30 cycles/second for a 4-minute cycle. RNA was extracted from tick homogenates using an automated MagMAX nucleic acid extraction kit and the associated instrument (ThermoFisher Scientific). Viral detection was determined using a previously described multiplex POWV-1/DTV qRT-PCR assay, and viral load was determined based on a standard curve generated by 10-fold dilutions of a known viral titer.^18^

## STATISTICAL ANALYSES

All statistical analyses and data visualizations were completed in GraphPad Prism v. 9.0.1. Comparisons of survivor and nonsurvivor group samples were analyzed by paired Student’s *t*-tests before combination of groups for data visualization. Viremia and weight change were analyzed by two-way analysis of variance (ANOVA) with the Tukey multiple comparisons test. Survival data were analyzed by Mantel–Cox and Gehan–Breslow–Wilcoxon tests. Analysis of viral diversity was determined by one-way ANOVA with the Tukey multiple comparisons test. Growth kinetics in cell culture were analyzed by paired Student’s* t*-tests, and in *I. scapularis*, they were determined by one-way ANOVA with the Tukey multiple comparisons test. Infection rates in *I. scapularis* were analyzed by a χ^2^ test.

## RESULTS

### Epidemiology and isolation of recent lethal human-derived POWV strains.

In 2020, a fatal case of POWV was reported in northcentral MN. The patient was a 67-year-old male with a history of organ transplantation and immunosuppressive medication. Symptoms included fever, headache, altered mental state, coma, neurological deficits, hydrocephalus, and seizure. Subsequently, in 2021, a fatal human case was reported in southeastern NYS. The patient was a 39-year-old healthy female, in whom symptom onset occurred shortly after a reported tick bite and included fever, altered mental state, headache, seizures, loss of coordination, tremors, muscle and joint pain, and gastrointestinal issues. The patient was diagnosed with encephalitis and died 6 days after hospitalization. Both patients died within a month of initial symptom onset. Neither patient had known travel histories outside of their respective state. Patient CSF and serum were collected from both cases for further diagnostic testing. Arboviral confirmatory clinical testing at MN and NYS departments of health determined the causative agent to be POWV through qRT-PCR, IgM serological assays, and/or plaque reduction neutralization test. Live virus was isolated from patient CSF on baby hamster kidney cells (BHK-21), passaged once, and subsequently confirmed through qRT-PCR. Full-genome sequencing was conducted, and each isolate was determined to be lineage 2 or DTV. The isolates hereafter will be referred to as DTV MN-PV320 (GenBank: OL695841.1) and DTV NY21-027 (GenBank: PP151210.1).

### DTV NY21-027 results in increased mortality in mice.

**Figure 1.**
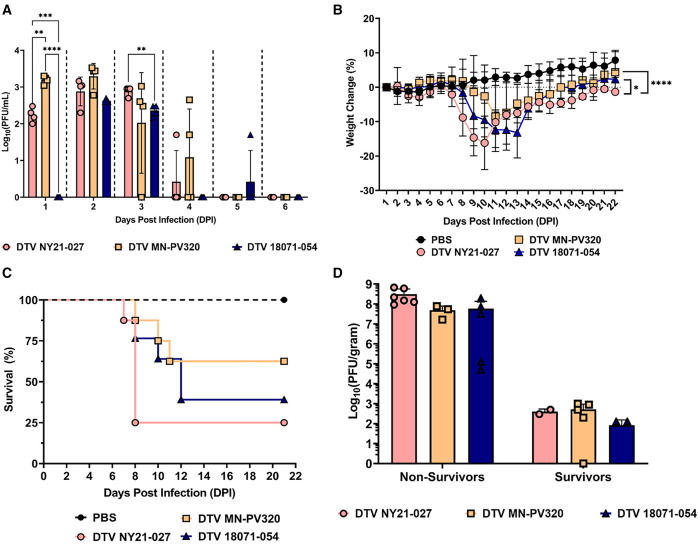
Virulence of human-derived deer tick virus (DTV) strains DTV NY21-027, DTV MN-PV320, and DTV 18071-054 (tick derived) in age-matched, mixed-sex C57BL/6 mice. All mice were inoculated through footpad injection with an equal amount of each respective strain or sterile phosphate buffered saline (PBS) as a control group. All data points represent overall mean plus or minus standard error of the mean (*n* = 8 per group). (**A**) Viremia kinetics reveal strain-specific differences at 1 and 3 days postinfection (DPI; two-way analysis of variance with the Tukey multiple comparisons test, 1 DPI: DTV NY21-027 vs. DTV 18071-054 [tick]). **P = 0.0040; ***P = 0.0004, DTV MN-PV320 vs. DTV 18071-054 (tick); **** P <0.0001, 3 DPI: DTV NY21-027 vs. DTV 18071-054 (tick). (**B**) Significant strain-specific differences were also noted in weight change during the course of infection (paired Student’s *t*-test, DTV NY21-027 vs. DTV MN-PV320). *P = 0.0123; ****P <0.0001, DTV NY21-027 vs. DTV 18071-054 (tick). (**C**) Mice infected with DTV NY21-027 experience mortality the earliest and at the highest rates (37.5–75.0% mortality; Mantel–Cox test, *P* = 0.0124; Gehan–Breslow–Wilcoxon test, *P* = 0.0075). (**D**) Viral loads in the brains of nonsurvivors (harvested at euthanasia between 7 and 11 DPI) and survivors (harvested at euthanasia on 21 DPI) of DTV-infected mice were also measured with plaque assay, revealing comparable viral loads between strains. PFU = plaque-forming unit.

To evaluate the virulence phenotypes of DTV MN-PV320 and DTV NY21-027, 6-week-old mixed-sex C57BL/6 mice were inoculated subcutaneously through the footpad with an equal amount (3 log_10_ PFU) of each respective strain to recapitulate the route and dosage delivered by tick feeding (*n* = 8 per strain).^38^ As POWV viremia is transient in mammalian hosts, serum was collected daily until 6 DPI and at 21 DPI or time of euthanasia to confirm infection and assess viral kinetics.^5^^,^^39^ After inoculation, all mice became productively infected by all POWV strains as measured by detectable serum viremia and/or brain titer (Figure 1). Viremia was equivalent between survivors and nonsurvivors in all DTV-inoculated groups (Figure 1A) (paired Student’s *t*-test, *P* >0.05). Significant strain-specific differences were detected at 1 and 3 DPI, with DTV NY21-027 and DTV MN-PV320 reaching significantly higher viral loads at 1 DPI compared with DTV 18071-054 (tick) and between DTV MN-PV320 and DTV 18071-054 (tick) at 3 DPI (Figure 1A). Of note, DTV MN-PV320 reached peak viremia at 1 DPI, whereas DTV NY21-027 reached peak at 3 DPI (Figure 1A).

Weight change and survival over the course of 21 days were assessed in DTV-infected mice, and viral loads in the brains were determined at euthanasia. All groups, independent of strain, had increased weight loss compared with the control group (Figure 1B). DTV NY21-027 resulted in significantly more weight loss at the earliest time points, with the largest weight losses documented between 7 and 10 DPI (Figure 1B). Infection by the regionally matched tick-derived DTV 18071-054 from NYS resulted in the next highest amount of weight loss measured between 8 and 14 DPI. DTV MN-PV320 infection resulted in the least significant weight loss compared with DTV NY21-027 and DTV 18071-054 (tick), with the most weight loss recorded between 9 and 11 DPI. All DTV-infected groups showed significant mortality compared with mock-treated, age-matched controls (Figure 1C). Notably, DTV NY21-027 resulted in a significantly higher mortality rate than DTV MN-PV320 (Figure 1C) (*n* = 6/8, 75.0% versus *n* = 3/8, 37.5%). DTV NY21-027 also caused mortality at the earliest time points (7 DPI), whereas mortality in DTV MN-PV320–infected mice did not occur until 9 DPI. Mortality was reported for DTV NY21-027 between 7 and 9 DPI, whereas DTV MN-PV320–associated mortality occurred between 9 and 11 DPI. All survivors (*n* = 2 for DTV NY21-027 and *n* = 5 for DTV MN-PV320) were euthanized at 21 DPI. Brain viral loads of each strain were then assessed to determine if mortality was associated with differences in viral load during neuroinvasive infection. Mice that succumbed to disease (nonsurvivors) had 7.60 ± 1.18 log_10_(PFU/g) at the time of death in the brain (days 7–11) and did not differ significantly between strains (Figure 1D). Survivors had 2.03 ± 1.06 log_10_(PFU/g) at 21 DPI in the brain after surviving neuroinvasive infection with viral loads also comparable between strains (Figure 1D).

### DTV NY21-027 and DTV MN-PV320 genotypes reflect geographic origin.

**Figure 2.**
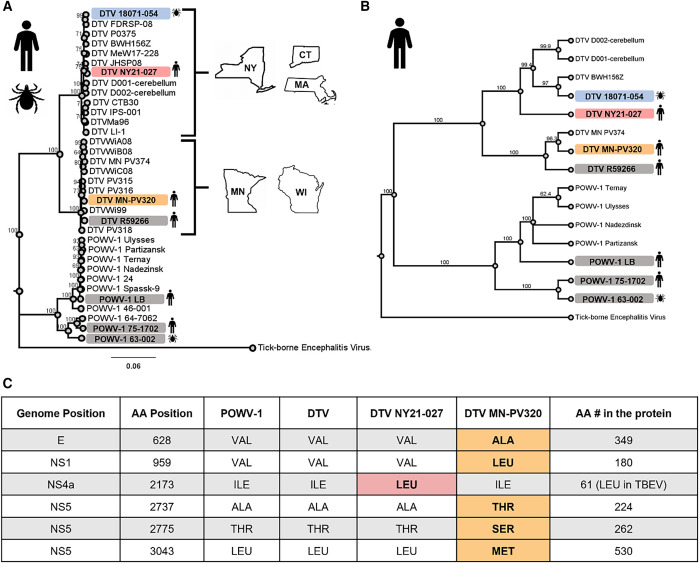
Maximum likelihood phylogeny of Powassan virus lineage 1 (POWV-1) and deer tick virus (DTV) strains based on full-genome nucleotide sequences with tick-borne encephalitis virus (TBEV; Oshima 08-As strain, Ab753012.1) included as an outgroup. Bootstrap values are displayed at each node (range: 63–100). Strains highlighted in gray are included in experimental results. (**A**) All strains cluster by lineage and geographic origin (POWV-1: Russia and northeastern United States; DTV: northeast and midwestern United States). (**B**) Maximum likelihood phylogeny of only human-derived POWV-1 and DTV strains. (**C**) Unique amino acid (AA) substitutions from recent human-derived DTV NY21-027 and DTV MN-PV320 compared with all publicly available POWV-1 and DTV strains. Unique substitutions are bolded and shaded dependent on the strain (orange: DTV MN-PV320; pink: DTV NY21-027). CT = Connecticut; MA = Massachusetts; MN = Minnesota; NY = New York; WI = Wisconsin.

To investigate if there were distinct genetic correlates that contribute to the observed mortality differences between DTV NY21-027 and DTV MN-PV320, each strain’s coding regions were compared with publicly available POWV-1 and DTV sequences (*n* = 441 strains, less than 10 available human-derived strains total). DTV NY21-027 shared close genetic similarity with human-derived strains from the northeastern United States, whereas DTV MN-PV320 clustered with sequences from the midwestern United States (Figure 2A and B). Overall, sequence identity between DTV MN-PV320 and DTV NY21-027 is 93.4% on the nucleotide level and 98.1% on the amino acid level, corresponding to 60-amino acid differences (Supplemental Table 2). The only unique amino acid substitution (i.e., not present in any other available POWV sequences) is I2173L (NS4a) in DTV NY21-027 (Figure 2C). Although this substitution is unique compared with all available POWV isolates, a leucine at position 2,173 is found at the consensus level across all TBEV subtypes. All other substitutions identified in DTV NY21-027 were shared with other strains or common substitutions specific to northeastern and midwestern foci (Supplemental Table 2). DTV MN-PV320 contains five unique substitutions located in the envelope (V628A), NS1 (V959L), and NS5 (A2737T, T2775S, and L3043M) (Figure 2C). Unlike DTV NY21-027, DTV MN-PV320 did not share any unique substitutions with TBEV.

### Viral population dynamics vary between lethal and nonlethal DTV infection.

Flaviviruses, like POWV, undergo stringent population bottlenecks as they transverse physical barriers, like the blood–brain barrier, during infection. We sought to quantify the within-host viral genetic diversity during POWV infection of mice. We identified iSNVs present throughout the viral genome by sequencing viral populations replicating in the brain and serum. For comparison, we also sequenced viral genomes from the challenge stock. For DTV NY21-027, 28 iSNVs shared between replicates were identified in mice that succumbed to infection, and 136 iSNVs were identified in mice that survived infection. All iSNVs remained below ∼45% frequency (cutoff: 2.0%). In contrast, five nonsynonymous iSNVs arose during DTV MN-PV320 infections, reaching high frequencies in mouse brains of animals that survived infection. Of note, these all arose from ∼2% frequency in the challenge stock to 100% in the mouse brains, and several amino acid substitutions were shared with DTV NY21-027 (Table 1). Amino acid substitutions present in DTV MN-PV320 and DTV 18071-054 (tick) were only detected in the survivors, with no changes present at the consensus level in the brains of nonsurvivors. The five substitutions (T2018A, I2173L, T2775S, P2895L, and E3037A) were present at 100% frequency in the DTV NY21-027 challenge stock, survivor, and nonsurvivor outputs. Notably, the unique I2173L substitution present in DTV NY21-027 emerged in both groups of survivors infected with DTV MN-PV320 and DTV 18071-054 (tick). At the minority level, no notable shared substitutions between nonsurvivors and survivors of any infection group were observed.

**Table 1:** Notable AA substitutions identified in viral populations derived from infected mouse brains of nonsurvivors and survivors of DTV strains DTV NY21-027, DTV MN-PV320, and DTV 18071-054

NT	AA	Protein	Input			Nonsurvivors			Survivors		
			DTV NY21-027	DTV MN-PV320	DTV 18071-054	DTV NY21-027	DTV MN-PV320	DTV 18071-054	DTV NY21-027	DTV MN-PV320	DTV 18071-054
6,052	2,018	NS3	ALA	THR	THR	ALA	THR	THR	ALA	ALA/THR	ALA
6,517	2,173	NS4a	LEU	ILE	ILE	LEU	ILE	ILE	LEU	LEU/ILE	LEU
8,324	2,775	NS5	THR	SER	THR	THR	SER	THR	THR	THR	THR/SER
8,683	2,895	NS5	LEU	PHE	LEU	LEU	PHE	LEU	LEU	PHE/LEU	PHE
9,110	3,037	NS5	GLU	ALA	ALA	GLU	ALA	ALA	GLU	GLU	ALA/GLU

AA = amino acid; DTV = deer tick virus; NS = nonsynonomous; NT = nucleotide. All substitutions were fixed at the consensus level of the DTV NY21-027 input for infection and resulting populations derived from serum and the brain.

**Figure 3.**
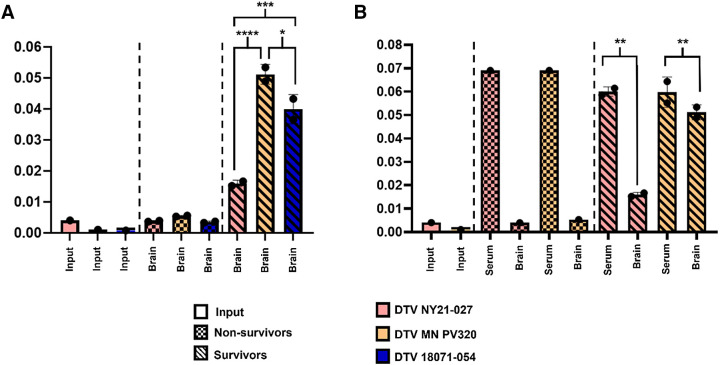
Minority variant analysis of input- (stock-), brain-, and serum-derived deer tick virus (DTV) NY21-027, DTV MN-PV320, and DTV 18071-054 (tick) viral populations. (**A**) Comparison of intrahost small nucleotide variants (iSNVs) present in brains of nonsurvivors compared with survivors of all strains as measured by the proportion of total minority variants (present in 2–49% of the population) in relation to the coding sequence genome size (∼10,300 kb). Data points represent mean plus or minus standard error of the mean (*n* = 1–2 per strain). Viral populations from all nonsurvivor brains of each strain were significantly less diverse than those that produced in survivor brains (one-way analysis of variance [ANOVA]). Of note, strain-specific differences between survivor brain viral populations were significant (one-way ANOVA with the Tukey multiple comparisons tests). *P = 0.0229 (DTV MN-PV320 vs. DTV 18071-054 [tick]); ***P = 0.0004 (DTV NY21-027 vs. DTV 18071-054 [tick]); ****P <0.0001 (DTV NY21-027 vs. DTV MN-PV320). (**B**) Comparison of iSNVs present in brains and matched sera of viral populations in nonsurvivors compared with survivors. Viral diversity is similar in serum independent of strain. Overall diversity in the brain was significantly lower than diversity in the corresponding serum in survivors (one-way ANOVA with the Tukey multiple comparisons test). **P = 0.0026 (DTV NY21-027 survivor brains and serum and DTV MN-PV320 survivor brains and serum).

In addition to iSNVs, the flavivirus quasispecies diversity is known to play a role in viral fitness. To further assess the relative diversity of the POWV populations replicating in mice, the viral nucleotide diversity (π) was determined for the nonsurvivors and survivors infected with each strain. Overall, π was similar in the challenge stocks of each strain compared with the survivor outputs (Figure 3A). Viral diversity in the brains of survivors, however, was higher across all strains (Figure 3A). Within the survivor groups, there were significant strain-specific differences, with the lowest diversity measured for DTV NY21-027 (Figure 3A). Viral diversity was also measured in matched serum samples of nonsurvivors and survivors after DTV NY21-027 or DTV MN-PV320 infection (Figure 3B). Serum diversity was similar among strains. For nonsurvivors, brain viral diversity was lower than diversity of virus derived from serum for both strains (Figure 3B). In the survivors, however, although viral diversity was still significantly lower in the brains infected with either strain, the difference between DTV NY21-027–infected serum and brains was significantly greater than the difference between DTV MN-PV320–infected serum and brains (Figure 3B).

### DTV NY21-027 displays a fitness deficit in mammalian cell culture.

**Figure 4.**
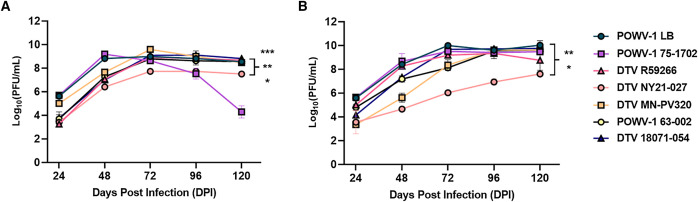
Growth kinetics of human-derived Powassan virus lineage 1 (POWV-1) and deer tick virus (DTV) strains compared with representative tick-derived strains in (**A**) baby hamster kidney (BHK-21) and (**B**) human neuroblastoma cells (SH-SY5Y). Data points represent mean plus or minus standard error of the mean (*n* = 3 per strain). DTV NY21-027 displayed a significant growth deficit in BHK-21 and SH-SY5Y. (**A**) Paired Student’s *t*-test. *P = 0.0362 (comparison with DTV R59266); *P = 0.0100 (comparison with DTV 18071-054 [tick]); **P = 0.0055 (comparison with DTV NY21-027, POWV-1 LB); **P = 0.0043 (comparison with POWV-1 63-002 [tick]); ***P = 0.0009 (comparison with DTV MN-PV320). (**B**) Paired Student’s *t*-test. *P = 0.0423 (comparison with DTV MN-PV320); **P = 0.0014 (comparison with DTV NY21-027, POWV-1 LB); **P = 0.0025 (comparison with POWV-1 75-1702); **P = 0.0077 (comparison with DTV R59266); **P = 0.0010 (comparison with POWV-1 63-002); **P = 0.0093 (comparison with DTV 18071-054 [tick]). DPI = days postinfection; PFU = plaque-forming unit.

To further investigate the differences in fitness between DTV NY21-027 and DTV MN-PV320, replicative fitness was assessed in mammalian cell culture and compared with other publicly available human-derived POWV strains: POWV-1 LB, POWV-1 75-1702, DTV R59266, and representative tick isolates of each lineage from NYS (Table 2). Viral infectivity and replication were assessed in vitro in BHK-21 and differentiated human neuroblastoma (SH-SY5Y) cells to recapitulate replication in small mammalian hosts in nature and infection of human brain cells. DTV NY21-027 displayed a significant growth deficit in both BHK-21 and SH-SY5Y, whereas DTV MN-PV320 displayed similar kinetics to all other strains (Figure 4). In BHK-21, DTV NY21-027 grew to significantly lower viral loads at all time points (Figure 4A). Although POWV-1 75-1702 displayed a decrease in viral load by 120 HPI, it was not significantly different compared with other strains. In SH-SY5Y, all strains displayed similar kinetics, except for DTV NY21-027, which again grew to significantly lower viral loads at all time points (Figure 4B). Of note, DTV MN-PV320 grew to lower viral loads at 48 HPI compared with all other strains, but the difference was not significant and was resolved by 72 HPI. Despite these variable kinetics, there were no observed differences in cytopathic effect, plaque morphology, or plaque size across strains.

**Table 2:** Human- and tick-derived POWV and DTV strain information

Strain Designation	Lineage	Location	Year	Source	Passage History	Accession No.
POWV-1 LB	1	Ontario, Canada	1958	Human	P10SM2V1B4	NC003687.1
POWV-1 75-1702	1	New York, USA	1975	Human	SM4B2	OP265689.1
DTV R59266	2	Ontario, Canada	1997	Human	P?B1	PP151211.1
DTV MN PV320	2	Minnesota, USA	2020	Human (CSF)	B2	OL695841.1
DTV NY21-027	2	New York, USA	2021	Human (CSF)	B2	PP151210.1
POWV-1 63-002	1	New York, USA	2018	*Ixodes cookei*	B2	OP265691.1
DTV 18071-054	2	New York, USA	2018	*Ixodes scapularis*	B2	OP265695.1

CSF = cerebrospinal fluid; DTV = deer tick virus; POWV = Powassan virus. Notably, recent human- and tick-derived strain stocks used were limited to a passage history of B2 (isolation and passage on BHK-21).

### Human-derived POWV strains exhibit comparable fitness in nymphal ticks.

**Figure 5.**
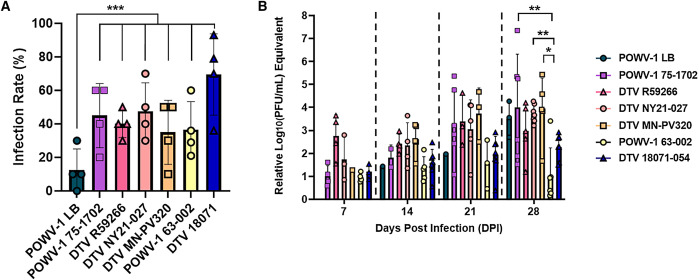
Infectivity and replication kinetics of human- and tick-derived Powassan virus lineage 1 (POWV-1) and deer tick virus (DTV) in experimentally infected *Ixodes scapularis*. (**A**) Data points represent each time point (*n* = 10 per time point per strain at 7, 14, 21, and 28 days postinfection [DPI]), with columns representing the overall mean plus or minus standard error of the mean. The only significant difference in infection rates was detected between POWV-1 LB and all other strains (χ^2^ test). ***P <0.001. (**B**) Columns represent mean plus or minus standard error of the mean of POWV-1– or DTV-positive nymphs at each time point (*n* = 1–7, *n* = 0 for POWV-1 LB at 7 DPI). Significant differences in overall viral load were only detected at 28 DPI between POWV-1 75-1702, DTV NY21-027, DTV MN-PV320, and POWV-1 63-002 (tick) (one-way analysis of variance with the Tukey multiple comparisons test). *P = 0.0127; **P = 0.0054; **P = 0.0098, respectively. PFU = plaque-forming unit.

Host cycling between vertebrate and invertebrate hosts is an important aspect of the flavivirus life cycle that imposes multiple unique selective pressures that can alter viral fitness in each respective host.^40^^,^^41^ To evaluate if the phenotypic variability observed in mice and mammalian culture correlated to altered replication in the tick host, infectivity and replication of the human-derived POWV strains were also determined in vivo in *I. scapularis* nymphs. Nymphal ticks were infected with POWV strains by immersion, a process that bypasses the blood-feeding process and recapitulates early infection and establishment of the virus.^33^^,^^37^ Infection rates of POWV-1 75-1702, DTV R59266, DTV NY21-027, DTV MN-PV320, POWV-1 63-002, and DTV 18071-054 were similar across all time points (Figure 5A). Only POWV-1 LB displayed significantly lower infection rates compared with all other strains (Figure 5A).

Viral load was also statistically similar between the human-derived strains and DTV 18071-054 (tick) through 28 DPI (Figure 5B). Similar to infectivity, DTV NY21-027 and DTV MN-PV320 exhibited comparable viral loads at all time points. The only significant differences were detected between POWV-1 75-1704, DTV NY21-027, and DTV MN-PV320 compared with tick-derived POWV-1 63-002 (Figure 5B). Despite not being significantly different at 14, 21, or 28 DPI, all human-derived strains, except for POWV-1 LB, had higher overall viral loads than those measured for tick-derived strains POWV-1 63-002 and DTV 18071-054.

Genome sequencing was also conducted for all strains from virus recovered from infected nymphal ticks at 28 DPI. Notably, there were no shared amino acid substitutions that emerged across all strains after maintenance in *I. scapularis*. Unique amino acid substitutions in DTV NY21-027 (I2173L) and DTV MN-PV320 (V628A, V959L, A2737T, T2775S, and L3043M) were all maintained in each respective strain.

## DISCUSSION

This study reports the first known comparison of two contemporary DTV isolates from fatal human cases derived from regionally distinct transmission foci in the United States. In comparison, most previous studies have relied on historic strains, primarily POWV-1 LB and DTV Spooner.^39^^,^^42^ These strains have long complex passage histories, constitute different lineages, and originate from distinct transmission foci from Canada and Wisconsin, respectively. Our work, therefore, provides a basis for beginning to understanding strain-specific virulence of recent low-passage DTV derived from geographically distinct regions with high case burdens.

Because POWV foci remain highly stable, strain variation likely occurs because of founder effects followed by unique stochastic and selective pressures resulting from host-specific adaptation, environmental changes that alter host physiology, or distinct transmission bottlenecks.^21^^–^^23^^,^^33^^,^^41^^,^^43^ Similar transmission and viral diversification patterns have occurred during TBEV spread throughout Eurasia. TBEV subtypes vary by ∼20% and ∼6% at the nucleotide and amino acid levels, respectively, with a proposed evolutionary rate of 1 × 10^−4^ substitutions per site per year.^27^^–^^29^^,^^44^^,^^45^ Subtype transmission occurs in highly focal patterns, like POWV, with ecological maintenance accomplished by multiple tick species, primarily *Ixodes ricinus* for European subtypes and *Ixodes persulcatus* for Far East subtypes.^46^ It is hypothesized that TBEV divergence occurred because of rare host-switching events followed by gradual stochastic evolution within these foci.^44^^,^^45^ Of note, TBEV divergence driven by host switching in nature subsequently resulted in diversification in clinical outcomes. The role of host-specific adaptation after these rare spillover events has also been observed for POWV across various tick genera, particularly in the United States, yet how this diversification could influence disease severity remains understudied.^33^^,^^45^ Notably, clinical differences between POWV-1 and DTV remain to be determined, despite following similar patterns of genetic divergence as the Far East and Siberian TBEV subtypes in Asia.^44^ Although genetic variance has been associated with differences in TBEV disease progression, other factors are also known to contribute to TBEV and POWV disease, including age, immune status, host genetics, and coinfections.^5^^,^^46^^,^^47^

Results of infections of C57BL/6 mice with DTV NY21-027 and DTV MN-PV320 support the hypothesis that geographic and genetic divergence can contribute significantly to variation in disease severity. Infections with these strains resulted in distinct differences in viremia kinetics, weight loss, mortality, and intrahost virus population dynamics. Viremia during the initial phases of infection was similar to previous studies that measured peak viremia between 1 and 3 DPI and total clearance from the serum by 5–6 DPI.^39^^,^^42^^,^^47^ Differences in viral loads in the serum between strains were moderate, but DTV MN-PV320 did reach peak viremia earlier. This suggests modest differences in replicative fitness or immune evasion mechanisms, but further work is required to elucidate the mechanism. Previous studies found that decreased viremia and accelerated clearance correlated to protection from lethal, neuroinvasive infection in collaborative cross mice.^48^ Conversely, infection with DTV NY21-027 resulted in increased weight loss and mortality at the earliest time points relative to DTV MN-PV320, suggesting differential virulence potential in human hosts. These results are generally consistent with the clinical presentations from the associated human cases. Despite both cases being fatal, the NYS patient was a healthy 39-year-old woman who experienced rapid progression of POWV acute illness. The MN case was in a 67-year-old patient with a history of immunosuppression who succumbed to infection, a clinical picture closer to previously reported cases. To date, reported human cases are primarily patients who are male, are 65 years old or older, and often have a history of comorbidities.^5^^,^^49^ Although there were no strain-specific differences in viral load in experimentally infected C57BL/6 mice, the observed differences in mortality in mice and disease presentation in the human cases support the notion that DTV NY21-027 may result in more severe disease because of mechanisms independent from viremia. Although these data are compelling, additional geographically distinct isolates should be tested in the future to further address the role of regional evolution in POWV disease.

To further explore the mechanisms contributing to increased mortality caused by DTV NY21-207, we sought to identify potential genetic correlates at consensus and minority levels and to experimentally assess phenotypes in natural (small mammals and ticks) and dead-end (human) hosts for POWV. Sequencing results revealed that DTV NY21-027 and DTV MN-PV320 exhibit high genetic similarity to regionally matched DTV strains. Strain- and region-specific amino acid substitutions were identified in both strains, but comparable virulence in mice and cell culture of DTV MN-PV320 suggests that substitutions present in this strain do not contribute to increased viral fitness during infection and likely emerged stochastically.^50^ DTV NY21-027, however, has a single discrete substitution relative to all publicly available POWV-1 and DTV strains. This substitution, I2173L, is located in the transmembrane domain region of the NS4a protein, with implications in protein stability, host membrane remodeling, and host factor recruitment.^51^ In dengue virus serotype 2 and Modoc virus, NS4a has also been implicated in the establishment of persistent infection through regulation of host cell autophagy pathways.^51^ These mechanistic roles could explain the observed decrease in overall viral fitness of DTV NY21-027 in mammalian and human cell culture systems as it may function to decrease host cell lysis and result in more persistent viral release over time. The potential role of I2173L in altered viral fitness was further supported by the maintenance of this substitution at the consensus level for DTV NY21-027, and most strikingly, its emergence in the brains of mice that survived infection with DTV MN-PV320. These data support the idea that I2173L is selected for during infection in mice, independent of strain genetic background or phenotype. Other emergent substitutions could complement the role of I2173L and were also present in the DTV NY21-027 input and viral brain outputs from DTV MN-PV320 survivors. These additional substitutions spanned NS3 and NS5 proteins that are primarily responsible for polyprotein cleavage and viral replication.^52^ NS5 encodes the methyltransferase and RNA-dependent RNA polymerase, which control the generation of diversity within the POWV quasispecies, an important element for host cycling for multihost viruses.^40^^,^^41^^,^^53^^,^^54^ These changes to NS5 could alter the methyltransferase activity and indirectly perturb polymerase fidelity, contributing to strain-specific differences in viral replication and diversity.^53^^–^^55^ Notably, just as DTV MN-PV320 acquired these NS3, NS4a, and NS5 substitutions during nonlethal infection, there were changes in viral diversity in the brains of these survivors similar to that observed in DTV NY21-027. It is also possible that these substitutions may not impact replicative fitness but could, instead, alter host immune activation, which can influence disease severity. Of note, the untranslated regions at the 5′ and 3′ end of the POWV genome were not included but are also known to influence transmissibility, immune evasion, and viral replication.^56^ Although most available POWV sequences lack these data, it is important to consider this for future work.

Because DTV is maintained in nature by *I. scapularis*, we also sought to understand if these strain-specific differences between DTV NY21-027 and DTV MN-PV320 were reflected in the invertebrate host. All POWV-1 and DTV human-derived strains displayed comparable infection rates and viral kinetics in *I. scapularis* experimentally infected by immersion. Similar to previous reports, there was no correlation between infection rates and replicative fitness, with all strains reaching peak viremia between 21 and 28 DPI.^33^ As DTV NY21-027 and DTV MN-PV320 were derived from brain-adapted populations, an observed fitness trade-off, which is commonly reported during host cycling between vertebrate and invertebrate hosts, was expected.^40^ Unexpectedly, all strains derived from human samples (CSF or brain tissue) displayed higher viral loads over time in *I. scapularis*, suggesting that selective pressures within the tick do not constrain mammalian-associated virulence evolution. Additionally, the maintenance of I2173L and the multiple unique substitutions identified in DTV MN-PV320 further suggest that maintenance in a tick host does not result in emergence of these genetic changes and is perhaps driven by the mammalian host. Despite these findings, there are potential limitations to these data, primarily the lack of a blood meal when infecting by immersion. Blood feeding is important for physiological changes in the tick and could result in altered tropism and immune landscapes for viral populations.^57^

## CONCLUSION

Overall, this study highlights the importance of POWV genetic diversity in clinical outcome. Notably, the potential for divergent, regionally distinct POWV phenotypes associated with variable clinical presentation could further complicate human case reporting, diagnostics, and treatment. Further isolation and characterization of POWV strains from human infections, fatal and nonfatal, could increase our understanding of POWV virulence and genetic correlates of disease. Future studies assessing representative, regionally distinct tick-derived strains would further complement this work and help track POWV phenotypic heterogeneity in North American transmission foci.

## Supplemental Materials

10.4269/ajtmh.24-0776 Supplemental Materials

## Data Availability

Full-genome sequences of deer tick virus (DTV) NY21-027, DTV MN-PV320, and DTV R59266 were submitted to the NIH National Center for Biotechnology Information GenBank (Accession nos. DTV NY21-027-PP151210.1, DTV MN-PV320-OL695841.1, and DTV R59266-PP151211.1). All other sequences used in this study are publicly available through GenBank (Table 2). All other datasets are contained within this publication.
