# Differential Virulence and Host-Specific Fitness of Regionally Distinct Human-Derived Powassan Virus Lineage 2 Strains

**DOI:** 10.4269/ajtmh.24-0776cor

**Published:** 2026-02-10

In the original research paper, “Differential Virulence and Host-Specific Fitness of Regionally Distinct Human-Derived Powassan Virus Lineage 2 Strains” by Rachel E. Lange, et al., 2025, (https://doi.org/10.4269/ajtmh.24-0776), an error was introduced prior to publication in the designation for DTV strain R59266. After publication, the authors were notified by the Centers for Disease Control and Prevention (the source of the virus isolate) that an error in the labeling of the stock tube existed, and that the virus strain was a variant of DTV Spooner rather than DTV strain R59266 (originally requested). The paper has since been corrected. The CDC and the authors deeply regret this error. Importantly, the original results and conclusions of the manuscript remain unchanged. A summary of the corrected data is shown below:

1. “Viral isolates POWV-1 LB and DTV Spooner were provided by the CDC.” (MATERIALS AND METHODS: page 107)
2. “To further investigate the differences in fitness between DTV NY21-07 and DTV MN-PV320, replicative fitness was assessed in mammalian cell culture and compared with other publicly available human-derived POWV strains: POWV-1 LB, POWV-1 75-1702, and representative tick isolates of each lineage from NYS and Wisconsin (Table 2).” (RESULTS: page 112)
3. “Infection rates of POWV-1 75-1702, DTV Spooner, DTV NY21-027, DTV MN-PV320, POWV-1 63-002, and DTV 18071-054 were similar across all timepoints (Figure 5A).” (RESULTS: page 112)
4.
  **Table:** 

Strain Designation	Lineage	Location	Year	Source	Passage History	Accession Number
POWV-1 LB	1	Ontario, Canada	1958	Human	P10SM2V1B4	NC003687.1
POWV-1 75-1702	1	New York, USA	1975	Human	SM4B2	OP265689.1
**DTV Spooner**	**2**	**Wisconsin, USA**	**2008**	***Ixodes scapularis***	**P?B1**	**HM440560.1**
DTV MN PV320	2	Minnesota, USA	2020	Human (CSF)	B2	OL695841.1
DTV NY21-027	2	New York, USA	2021	Human (CSF)	B2	PP151210.1
POWV-1 63-002	1	New York, USA	2018	*I. cookei*	B2	OP265691.1
DTV 18071-054	2	New York, USA	2018	*Ixodes scapularis*	B2	OP265695.1
  (TABLE 2 – Row 3: page 112)
5. “**FIGURE 4.** Growth kinetics of human-derived Powassan virus lineage 1 (POWV-1) and deer tick virus (DTV) strains… (**A**) Paired Student’s *t*-test. *P = 0.0362 (comparison with DTV Spooner)…(**B**) **P = 0.0077 (comparison with DTV Spooner)…” (Figure 4: page 113)
6. “Full genome sequences of deer tick virus (DTV) NY21-027 and DTV MN-PV320 were submitted to the NIH National Center for Biotechnology Information GenBank (Accession Nos. DTV NY21-027-PP151210.1 and DTV MN-PV320-OL695841.1)” (DATA AVAILABILITY: page 115)
7. Figure 2A–2B: In phylogenetic tree “DTV R59266” changed to “DTV Spooner”.
  Figure 4A–4B: In replication kinetics legend “DTV R59266” changed to “DTV Spooner”.
  Figure 5A–5B: In infectivity bar graph and replication kinetics legend “DTV R59266” changed to “DTV Spooner”. (Figures 2, 4, and 5: pages 111 and 113)

