# Supplementary material for: Differential Virulence and Host-Specific Fitness of Regionally Distinct Human-Derived Powassan Virus Lineage 2 Strains
Source: Am J Trop Med Hyg. 2025 May 13;113(1):106–16. doi: 10.4269/ajtmh.24-0776 (PMC12225551; doi:10.4269/ajtmh.24-0776)
Supplement: Supplemental Materials [file tpmd240776.SD1.pdf]

Supplemental Table 1

Powassan virus whole genome sequencing primers. F and R are defined as forward and reverse respectively. Gene fragments include regions spanning the structural proteins capsid, pre-membrane, and envelope (CprME), nonstructural proteins (NS), and flanking untranslated regions (UTR)

**Primer**	**Coverage**	**Region**	**Sequence**
Frag 1 F	5'UTR	CprME	agattttcttgcacgtgtgtgcgg
Frag 1 R	2620c		tggtggcagctgcatttctat
Frag 2 F	2116	NS1-NS3	cagcagtggtttcagaaaggcagt
Frag 2 R	4792c		acgtcttcacgcacatccgccca
Frag 3 F	3107	NS2a-NS3	actgcacatggccagcaagtcaca
Frag 3 R	5581c		tcactcactatggctcctttgga
Frag 4 F	4423	NS3-NS4b	atggggaacttgcacttgacagag
Frag 4 R	7197c		gtgggcgtcgctcctacc
Frag 5 F	6319	NS4a-NS5	gaactggtcacgttcagaagcccc
Frag 5 R	9300c		tccatgtaccgaaggatctgctct
Frag 6 F	8566	NS5-3-UTR	tggggcagctatcgcactc
Frag 6 R	3'UTR		ggctcaccaggagttaggccgttt

Supplemental Table 2

Amino acid differences between human-derived deer tick virus (DTV) strains DTV NY21-027 and DTV MN-PV320. DTV NY21-027 contains a singular substitution unique to this strain while DTV MN-PV320 contains five. Most differences were either shared with other publicly available strains or were regionally specific (northeastern and midwestern United States foci). Substitutions were further defined by a note indicating it's relative uniqueness among all available POWV-1 and DTV strains (*Regional* – substitution is shared among strains isolated from the same region or foci, *Shared with other strains* – substitution is shared with one or more strains, *Lineage-1-like* – substitution is shared among POWV-1 strains, *Unique to X* – substitution is only present in the specified strain)

Amino Acid Position	DTV NY21	DTV MN PV320	Note
26	PRO	LEU	Shared with other strains
29	SER	GLY	Regional
78	ARG	LYS	Shared with other strains
110	ALA	THR	Regional
121	LYS	ARG	Regional
453	ASN	GLY	Lineage-1-like
483	ASN	SER	Shared with other strains
628	VAL	ALA	Unique to DTV MN-PV320
716	VAL	ILE	Lineage-1-like
764	VAL	ALA	Regional
953	ARG	LYS	Shared with other strains
959	VAL	LEU	Unique to DTV MN-PV320
995	ALA	THR	Shared with other strains
1139	THR	VAL	Lineage-1-like
1180	ARG	GLY	Shared with other strains
1188	ILE	PHE	Shared with other strains
1221	ILE	THR	Shared with other strains

1225	VAL	THR	Shared with other strains
1227	ARG	LYS	Regional
1253	PHE	LEU	Regional
1315	SER	ASN	Regional
1353	GLY	ARG	Shared with other strains
1356	ARG	HIS	Shared with other strains
1458	ALA	VAL	Regional
1484	PHE	LEU	Shared with other strains
1487	ALA	THR	Regional
1504	LYS	SER	Shared with other strains
1522	VAL	LEU	Shared with other strains
1637	VAL	ILE	Shared with other strains
1667	GLU	ASP	Shared with other strains
1672	ILE	VAL	Regional
1749	LEU	SER	Regional
1847	ILE	VAL	Shared with other strains
1916	ASN	HIS	Shared with other strains
2018	ALA	THR	Shared with other strains
2044	ALA	GLU	Shared with other strains
2173	LEU	ILE	Unique to DTV NY21-027
2179	VAL	ILE	Shared with other strains
2207	LEU	MET	Shared with other strains
2258	LEU	VAL	Regional

2285	ARG	GLY	Shared with other strains
2339	LYS	ARG	Regional
2414	VAL	ILE	Regional
2438	VAL	ILE	Regional
2462	VAL	ILE	Shared with other strains
2473	GLU	ALA	Shared with other strains
2532	THR	SER	Shared with other strains
2563	MET	ILE	Shared with other strains
2613	LYS	ARG	Regional
2652	VAL	ALA	Regional
2737	ALA	THR	Unique to DTV MN-PV320
2775	THR	SER	Unique to DTV MN-PV320
3037	GLU	ALA	Shared with other strains
3043	LEU	MET	Unique to DTV MN-PV320
3161	LYS	ARG	Shared with other strains
3290	LEU	PHE	Lineage-1-like
3303	MET	ILE	Shared with other strains
3339	ASN	ASP	Lineage-1-like
3342	MET	THR	Lineage-1-like
3391	GLU	ASP	Lineage-1-like
